# An Improved Parameter-Estimating Method in Bayesian Networks Applied for Cognitive Diagnosis Assessment

**DOI:** 10.3389/fpsyg.2021.665441

**Published:** 2021-05-24

**Authors:** Ling Ling Wang, Tao Xin, Liu Yanlou

**Affiliations:** ^1^School of Educational Science, Shenyang Normal University, Shenyang, China; ^2^Faculty of Psychology, Beijing Normal University, Beijing, China; ^3^Collaborative Innovation Center of Assessment toward Basic Education Quality, Beijing Normal University, Beijing, China; ^4^China Academy of Big Data for Education, Qufu Normal University, Qufu, China

**Keywords:** Bayesian Networks, ideal response pattern, cognitive diagnostic model, parameter estimating method, cognitive diagnostic assessment

## Abstract

Bayesian networks (BNs) can be employed to cognitive diagnostic assessment (CDA). Most of the existing researches on the BNs for CDA utilized the MCMC algorithm to estimate parameters of BNs. When EM algorithm and gradient descending (GD) learning method are adopted to estimate the parameters of BNs, some challenges may emerge in educational assessment due to the monotonic constraints (greater skill should lead to better item performance) cannot be satisfied in the above two methods. This paper proposed to train the BN first based on the ideal response pattern data contained in every CDA and continue to estimate the parameters of BN based on the EM or the GD algorithm regarding the parameters based on the IRP training method as informative priors. Both the simulation study and realistic data analysis demonstrated the validity and feasibility of the new method. The BN based on the new parameter estimating method exhibits promising statistical classification performance and even outperforms the G-DINA model in some conditions.

## Introduction

Cognitive diagnosis models (CDMs) are psychometric models developed mainly to assess students' specific strengths and weaknesses on a set of finer-grained skills or attributes within a domain. Researchers have developed many kinds of CDMs for cognitive diagnostic assessment (CDA), including the rule space model (Tatsuoka, 1985), the attribute hierarchy model (AHM, Leighton et al., 2004), the deterministic inputs noisy and gate model (DINA, Junker and Sijtsma, 2001), the Deterministic Input, Noisy Output “Or” gate model (DINO; Templin and Henson, 2006), the Log-Linear Cognitive Diagnosis Model (LCDM, Henson et al., 2009), the General Diagnostic Model (GDM, von Davier, 2005), the G-DINA model (de la Torre, 2011), and so on. In addition to these traditional CDMs, some researchers also proposed to use Bayesian networks (BNs, Pearl, 1988) in CDA (Mislevy, 1995; Mislevy et al., 1999; Almond et al., 2007, 2015; Wu, 2013; Levy and Mislevy, 2017). These literatures have documented the efficiency of BN used for diagnostic assessment, especially in the construction of large-scale evaluation system (e.g., Steinberg et al., 2003; Levy and Mislevy, 2004; Almond et al., 2015).

In BNs for CDA, the observable variables are the item responses and the latent variables are the knowledge states that can be treated as missing data. In Bayesian Network, like the traditional CDMs, the parameter estimation is also needed. The parameter estimation algorithms include the Markov Chain Monte Carlo (MCMC) method, the expectation maximization (EM) method, and the gradient descent (GD) learning method. In educational assessment, the MCMC method was used extensively (Wu, 2013; Almond et al., 2015; Levy and Mislevy, 2017), whereas the EM and the GD algorithms are rarely used. Some researches (Lauritzen, 1995; Russell et al., 1995; Korb and Nicholson, 2010) have described the algorithms. The EM algorithm is an iterative method to find (local) maximum likelihood estimates of parameters in BNs, in which the model depends on unobserved latent variables. EM iteration alternates between performing an expectation (E) step, which uses regular BN inference with the existing BN to compute the expected value of all the latent variables (missing data), and a maximization (M) step, which computes parameters maximizing the log-likelihood of BN given the resulting extended data (i.e., original data plus the expected value of missing data). These parameter estimates are used to determine the distribution of the latent variables in the next E step. The GD learning method tries to minimize the objective function of the negative log likelihood to determine the BN parameters. This method can find a better BN by using BN inference to calculate the direction of the steepest gradient and to change the parameters to follow the steepest direction of the gradient (i.e., maximum improvement). Actually, it uses a much more efficient approach than always taking the steepest path, by taking into account its previous path, which is why it is called the conjugate gradient descent (Norsys Inc., 2004). However, both the EM and the GD methods are sensitive to the initial condition and liable to be trapped in a local optimization. Moreover, there is a fundamental issue with using the EM and the GD algorithms in BN. In educational applications, the variables (both proficiency variables and observable outcomes) are usually ordinal, and the parameters are monotonic (greater skill should lead to better item performance). However, most parameter estimation algorithms do not force monotonicity constraints on the parameters. In the case when informative priors are available, they might support to keep the estimates from violating the monotonicity assumptions (von Davier and Lee, 2019). Almond (2015) describes a flexible parameterization for conditional probability tables based on item response theory (IRT) that preserves monotonicity and extends the EM algorithm to a generalized EM algorithm. However, this method needs to map each configuration of parent variables to an effective theta, a point on a latent normal scale, and calculate the conditional probability tables using the IRT model. That is to say, this method needs to introduce other models to assist the parameterization.

Besides the above CDMs, researchers also proposed to use machine learning algorithms for CDA, such as neural networks (NNs, Gierl et al., 2008; Shu et al., 2013; Cui et al., 2016), and support vector machines (SVMs, Liu and Cheng, 2018). Gierl et al. (2008) were the first to propose using the ideal response patterns (IRPs) and the corresponding attribute profiles constructed in the cognitive model of the CDA as the training data of NNs. The ideal response patterns are the students with the knowledge states (attribute profiles) to answer the questions with no slipping and guessing errors. The attribute profiles are the entire possible attribute combinations in the cognitive model. If there are K attributes in the test and the attributes are independent, then the number of the attribute profiles is 2^K^, while in the AHM, the number is < 2^K^ due to the hierarchical relationship of the attributes. Shu et al. (2013) also used this approach to train the NN and investigated its performance in small samples. They pointed out that, “In doing this all items are assumed to behave in the same way and so no differentiation is made between items with respect to item quality. A limitation of the use of a NN analysis with IRP is that all ‘calibration' of the model is based on connections between response patterns and attribute patterns calibrated in the training process, no empirical information (i.e., students' response patterns) is used to influence the procedure.” Thus, they proposed to combine the NN approach using IRP and a MCMC estimation algorithm or a Joint Maximum Likelihood Estimation (JMLE) algorithm. Inspired by this research, we propose to use the IRP data to estimate the BN parameters first, then continue to estimate the BN parameters by EM or GD methods, taking the previously estimated parameters as informative priors. The introduction of IRP into the EM or GD method can overcome the inaccurate estimation limitation caused by the violations of the monotonicity constraints. This combination also provides suitable starting values to continue the EM or GD estimation to overcome the local optimality problem. On the other hand, this method can also improve the performance of the IRP method without empirical information. And comparing to the solution in Almond (2015), we propose to apply the information contained in each CDA (IRP data) for the initial parameterization and do not need to introduce other models to assist the parameterization.

To demonstrate the effectiveness of our proposed approach, we performed a simulation study comparing the parameter estimating algorithms in BN, including the EM method, the GD method, the IRP training method, and the combinations of IRP and EM or GD methods. We considered the classification rates as the performance indicators of the algorithms and the G-DINA model's diagnostic classification performance as the evaluation criterion. Also, we carried out a real data analysis to demonstrate the validity of the proposed method.

Regarding the simulation method, usually various CDMs are adopted as the data-generating models. The simulation data is of great value in verifying the properties of the models. However, there are also some limitations in the CDM-based data. Real data do not necessarily conform to the assumptions of the CDMs. Using a CDM to process the data conforming to the same model assumption may overrate the properties of the model. Thus, other data-generating methods are needed. Wu (2013) proposed to generate data based on BNs, but the parameters of BNs were determined based on traditional CDMs, and the patterns of the simulated data by the BNs were essentially the same as the CDM-generated data. This research introduced a new simulation data generating method by using BNs to generate data with the empirical data's pattern and these simulated data do not necessarily satisfy the G-DINA assumption. To evaluate the BN and G-DINA methods more objectively, we designed the simulation study using both the G-DINA model and the BN model to generate data.

## Overview of the Cognitive Diagnostic Models and Bayesian Networks

### The Cognitive Diagnostic Model

The G-DINA model is a general CDM and can be converted to the constrained CDMs by setting appropriate constraints (de la Torre, 2011). According to de la Torre (2011) notation, for a test with *J* items and *K* attributes, let $K_{j}^{*}$represent the number of required attributes for item *j*, and $\alpha_{lj}^{*}$ be the reduced attribute vector whose elements are the required attributes for item *j*. Then, the item response function of the G-DINA model is expressed as follows:

$$\begin{array}{l} P(\alpha_{lj}^{*})=\delta_{j0}+\sum_{k=1}^{K_{j}^{*}}\delta_{jk}\alpha_{lk}+\sum_{k^{\prime }=k+1}^{K_{j}^{*}}\sum_{k=1}^{K_{j}^{*}-1}\delta_{jkk^{\prime }}\alpha_{lk}\alpha_{lk^{\prime }}...\\ \;+\delta_{j12...K_{j}^{*}}\prod_{k=1}^{K_{j}^{*}}\alpha_{lk} \end{array}$$

Where δ_*j*0_ is the intercept for item *j*; δ_*jk*_ is the main effect due to α_*k*_; $\delta_{jkk^{\prime }}$ is the interaction effect due to α_*k*_ and $\alpha_{k^{\prime }}$; and $\delta_{j12...K_{j}^{*}}$ is the interaction effect due to $\alpha_{1}...\alpha_{K_{j}^{*}}$. This function is the identity link function and the other two link functions can also be used to express the G-DINA model according to de la Torre (2011), namely, the logit link and the log link.

### The Bayesian Networks

Bayesian networks (BN, Pearl, 1988) are a notation for expressing the joint distribution of probabilities over both observed and latent variables. They are used to represent knowledge in an uncertain domain and have been successfully applied in computer science, especially in artificial intelligence. A BN consists of a directed acyclic graph (DAG) to represent their structure and a corresponding set of conditional probability distributions to represent the parameters (Culbertson, 2016; Hu and Templin, 2019). In this graph, each node represents a random variable and each edge constitutes the probabilistic dependence relationship among the variables represented by the two nodes that are joined. Each pair of connected nodes has a directed edge flowing from a “parent” node to a “child” node. A conditional probability distribution is specified for each node, given its parent nodes. For discrete random variables, this conditional probability is described with conditional probability tables (CPTs). The structure of BNs effectively reflects the conditional independent relationship between the variables. According to the conditional independent relationship, a joint probability distribution is decomposed into a product of a series of conditional distributions, which reduces the number of parameters required to define the joint probability distribution of the variables, and to compute the posterior probabilities efficiently, given the data.

**Figure 1:**
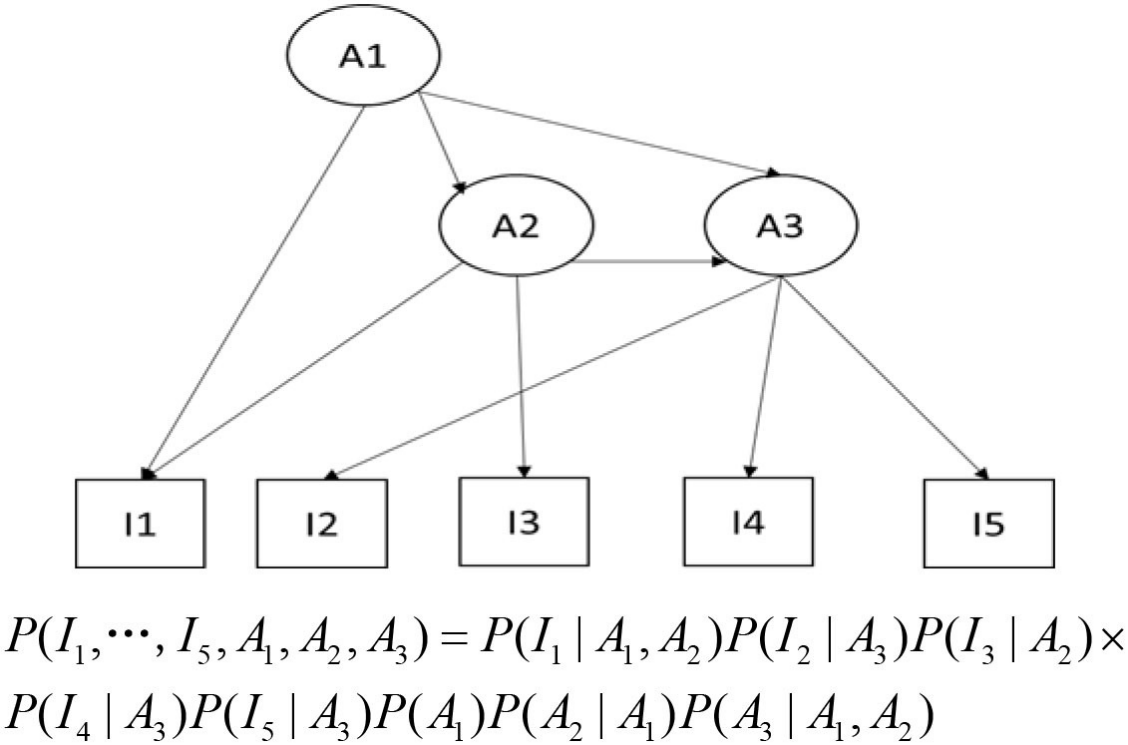
A Bayesian network applied for cognitive diagnosis.

A BN applied for cognitive diagnosis is shown in Figure 1. In Figure 1, the item nodes (observed variables) can be connected to the attribute nodes (hidden variables), similar to the Q-matrix that represented relationships between attributes and items in traditional CDMs (Tatsuoka, 1983; Almond, 2010). The attribute nodes can also be connected to each other, denoting the attribute hierarchical relationships. According to the specific structure of the BN, the joint probability distribution of all the variables can be factorized into a product of a series of conditional probabilities. Once the structure of the BN has been determined, we need to specify these parameters in the BN first in order to make an inference, i.e., specify the conditional probabilities and the marginal probabilities. If all the nodes of a discrete BN are fully observed in a sample, the CPTs can be updated via a simple counting algorithm. This is the case when we train the BN using the IRP and the corresponding attribute profile data. If there are missing values or latent variables, then the CPTs can be calculated using the EM algorithm, the GD method, or the MCMC method.

After obtaining the structure and parameters of the BN, we can use the BN to predict the students' knowledge state by probability inference. According to the Bayesian Theorem, the probability inference is when the posterior probability of the hidden variables (attributes) is calculated using the values of the observed variables (e.g., item response) as input. This process is also called network propagation or belief updating, which can be realized by a number of algorithms, such as the message passing algorithm (Pearl, 1988), or trees of cliques (Lauritzen and Spiegelhalter, 1988; Jensen, 1996).

## Methods

### Simulation Design

The study presented in this paper compared several parameters estimating methods in Bayesian networks for cognitive diagnosis. We conducted a simulation study to evaluate the performance of different parameter estimating methods. The Q-matrix (see Table 1) with 30 items and 5 attributes in de la Torre (2011) was adopted. The interconnections between attributes and test items in the Q-matrix could also be represented in a BN graph in which the arcs connecting two nodes denoted the associations between the corresponding items and the attributes.

**Table 1:** The Q-Matrix of the simulation study.

	**A1**	**A2**	**A3**	**A4**	**A5**		**A1**	**A2**	**A3**	**A4**	**A5**
I1	1	0	0	0	0	I16	0	1	0	1	0
I2	0	1	0	0	0	I17	0	1	0	0	1
I3	0	0	1	0	0	I18	0	0	1	1	0
I4	0	0	0	1	0	I19	0	0	1	0	1
I5	0	0	0	0	1	I20	0	0	0	1	1
I6	1	0	0	0	0	I21	1	1	1	0	0
I7	0	1	0	0	0	I22	1	1	0	1	0
I8	0	0	1	0	0	I23	1	1	0	0	1
I9	0	0	0	1	0	I24	1	0	1	1	0
I10	0	0	0	0	1	I25	1	0	1	0	1
I11	1	1	0	0	0	I26	1	0	0	1	1
I12	1	0	1	0	0	I27	0	1	1	1	0
I13	1	0	0	1	0	I28	0	1	1	0	1
I14	1	0	0	0	1	I29	0	1	0	1	1
I15	0	1	1	0	0	I30	0	0	1	1	1

The attribute patterns were generated in two different methods. The first method sampled attribute patterns from a uniform distribution with probability *1/2*^*K*^ of every possible value. In the second method, the discrete attribute pattern α was linked to an underlying multivariate normal distribution, *MVN (0*_*K*_, Σ*)*, with covariance matrix Σ, structured as

$$\begin{array}{l} \sum \text{=}\left(\begin{array}{cc} 1 & \rho \\ ...\\ \rho & 1 \end{array}\right) \end{array}$$

and ρ = 0.5. Let θ_*i*_ = (θ_*i*1_, θ_*i*2_, …, θ_*iK*_) denote the *K*-dimensional vector of latent continuous scores for examinee *i*. The attribute pattern α_*i*_ = (α_*i*1_, α_*i*2_, …, α_*iK*_) was determined by

$$\alpha_{ik}=\{\begin{array}{c} 1,if\theta_{ik}\geq \Phi^{-1}(\frac{k}{K+1})\\ 0,\text{otherwise} \end{array}$$

The item response data that were used in our experiments were generated using the G-DINA model.

The parameter setting include two conditions, one is that both the *g* and *s* parameters in each item are fixed at 0.2, the other is a mixed test with high-discriminating items and low-discriminating items (10 items with *g* = 0.3, *s* = 0.3; 10 items with *g* = 0.2, *s* = 0.2; and 10 items with *g* = 0.1, *s* = 0.1), which is more realistic in practice. The success probabilities of the examinees who master none and all attributes [i.e., P(0) and P(1)] were fixed as *g* and 1 – *s*, and the success probabilities of examinees with other attribute patterns were randomly generated from the distribution of Unif [P(0), P(1)] with monotonic constraint. All the above simulated data were generated by CDMs. We also used BNs to generate the data. When using BNs to generate the simulated data, the BN parameters need to be determined first because these parameters reflect the patterns behind the generated simulation data. Two datasets were used to estimate the BN parameters (i.e., the CPTs), including the simulated data previously generated by G-DINA, and the fraction subtraction dataset. The fraction subtraction dataset contains 536 students, 15 items, and 5 attributes, and the Q-matrix was defined as given by de la Torre (2009). Two BN models were constructed to process the two datasets and the parameters of the BN models were estimated based on the two datasets using IRP–EM method. Then, we used the two BN models to generate two types of simulated datasets. The patterns behind the two sets of simulated data reflect the patterns of the two sets of data previously used to estimate the parameters. In other words, the BN-generated data not only reflect the pattern of the G-DINA simulated data, but also the pattern of the real test data.

Accordingly, four types of datasets were simulated by CDMs (two level parameters × two different attribute profile sampling method) and two types of datasets were simulated by BNs. Each condition had two sample sizes of 500 and 1,000. And all the simulations are repeated 30 times to compute the average classification rate and the standard error.

### The Realization of the IRP–EM and IRP–GD Methods

**Figure 2:**
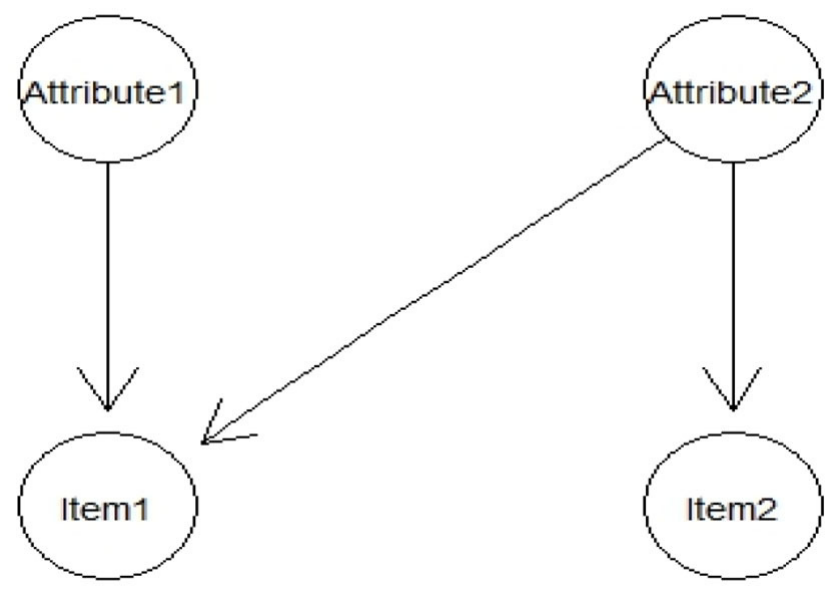
A simple Bayesian network applied for cognitive diagnosis.

The BN and G-DINA models were applied to analyze the data. The parameter estimating method of BNs includes the EM method, the GD method, the IRP training method, and the combinations of IRP with EM or GD methods. The classification rates are considered as the performance indicators of the algorithms and the diagnostic classification performance of the G-DINA model is considered as the evaluation criterion. When using IRP and the corresponding attribute profiles to train the BN, the CPT of the BN can be obtained through counting the observed frequencies in the IRP training dataset using algorithms (Neapolitan, 2004; Lee and Corter, 2011). A sample BN based on the problem of diagnosing attributes is shown in Figure 2. Table 2 presents the ideal response pattern and the corresponding attribute profiles; Table 3 shows the respective CPTs through counting the observed frequencies in the training dataset.

**Table 2:** The ideal response pattern and the corresponding attribute profiles.

	**Attribute 1**	**Attribute 2**	**Item 1**	**Item 2**
Case 1	1	1	1	1
Case 2	1	0	0	0
Case 3	0	1	0	1
Case 4	0	0	0	0

**Table 3:** The conditional probability table of the Bayesian network.

**P (Attribute1)**		**P (Attribute2)**	
**Attribute 1** **=** **1**	**Attribute 1** **=** **0**	**Attribute 2** **=** **1**	**Attribute 2** **=** **0**
2/4	2/4	2/4	2/4
**P (Item 1|Attribute 1, Attribute 2)**			
**Attribute 1**	**Attribute 2**	**Item 1** **=** **1**	**Item 1** **=** **0**
1	1	1	0
1	0	0	1
0	1	0	1
0	0	0	1
**P (Item 2|Attribute 1, Attribute 2)**			
**Attribute 1**	**Attribute 2**	**Item 2** **=** **1**	**Item 2** **=** **0**
1	1	1	0
1	0	0	1
0	1	1	0
0	0	0	1

The example with all the results presented above illustrated the BN training process using the IRP information. Two different types of IRP data were computed based on the DINA model and the DINO model representing the non-compensatory model and the compensatory model. After the BN was trained based on the IRP data, the GD or EM algorithm were used to estimate the parameters of BN treating the IRP parameters as priors. Generally, the DINA–IRP data were used first and when the GD or EM algorithms were trapped in the local optimality, the algorithms transfer to use the DINO–IRP data to train the BN model and continue to estimate the final parameters. No matter which IRP was used to train the BN, they provided the suitable starting values for the EM or the GD method and this avoided the algorithms to be trapped in the local optimality. The implementation of the parameter estimation algorithm is realized by Netica (www.norsys.com), a professional Bayesian network software. In this software, the IRP and the corresponding attribute patterns information is first entered into the constructed BN structure and the prior of the parameters in BN is obtained. After that, it continues to incorporate empirical data to update the BN posterior parameters based on the EM or GD algorithm.

## Results

We evaluated the performance of each algorithm under the conditions mentioned in the simulation design, using the average attribute classification rate (AACR) and the pattern classification rate (PCR) as the performance index for each condition. The standard error of each classification rate indicator is computed to evaluate the consistency of the methods. From Table 4, we could observe that in each condition, the PCRs of BNs based on EM or GD method were very low because the monotonic constraint was not satisfied. When the IRP training method was applied to estimate the parameters of BNs, the PCR was improved to some extent but still very low. In all these datasets, the effect of sample size on accuracy was not straightforward. When we used the IRP data to estimate the BN parameters first and continue to estimate the BN parameters by EM or GD methods by regarding the previously estimated parameters as informative priors, the PCRs of the BNs based on these two methods were significantly improved compared to the former three methods. The PCR of BN based on the combination of IRP and GD methods (denoted as BN–IRP–GD method) was higher than the BN based on the combination of IRP and EM method (denoted as BN–IRP–EM method) in these G-DINA datasets. The sample size had a certain influence on the accuracy rate, especially in the four G-DINA datasets processed by the IRP–EM method. The performance gap between the 1,000 and 500 samples of the other conditions was not so high. When comparing with the G-DINA analysis, in the four datasets generated by G-DINA, the PCR of BN–IRP–GD method was a little higher than the PCR of G-DINA if the attribute patterns of the students conformed to the uniform distribution, and the PCR of G-DINA was higher than the BN–IRP–GD method if the attribute patterns of the students conformed to the multivariate normal distribution.

**Table 4:** The PCR of BNs and G-DINA from the data generated by G-DINA.

***gs* Level**	**Distribution**	**Sample size**	**EM_BN**	**GD_BN**	**IRP_BN**	**IRP_EM_BN**	**IRP_GD_BN**	**G-DINA**
*g*=*s*=0.2	Uniform	1,000	0.196	0.162	0.320	0.611	0.627	0.622
		500	0.138	0.091	0.348	0.577	0.598	0.593
	Mvnorm	1,000	0.064	0.013	0.374	0.647	0.654	0.698
		500	0.112	0.084	0.344	0.627	0.641	0.681
Mixed *gs* (0.3, 0.2, 0.1)	Uniform	1,000	0.076	0.024	0.197	0.536	0.574	0.567
		500	0.002	0.000	0.190	0.471	0.536	0.533
	Mvnorm	1,000	0.036	0.038	0.263	0.582	0.644	0.677
		500	0.006	0.000	0.184	0.579	0.590	0.629

From Table 5, the results of AACR were similar to the PCR results. The AACRs of BNs based on EM or GD methods were very low and it was improved by training the BNs with the IRP data. The AACRs of BN–IRP–EM and BN–IRP–GD methods were improved further and the best result was achieved through the BN–IRP–GD method. When comparing with the G-DINA analysis, in the four datasets generated by G-DINA, the AACR of BN–IRP–GD method was a little higher than the AACR of G-DINA if the attribute patterns students conformed to the uniform distribution, and the AACR of G-DINA was higher than that of the BN-IRP-GD method if the attribute patterns of the students conformed to the multivariate normal distribution. We have also evaluated the consistency of the BN–IRP–EM method, the BN-IRP-GD method and the G-DINA method through standard errors of the PCR and AACR in Tables 6, 7. The standard errors of the AACR were lower than that of the PCR. Most conditions are lower than 0.01. The BN–IRP–GD method and the BN–IRP–EM method have the similar level of standard errors as the G-DINA method in all conditions.

**Table 5:** The AACR of BNs and G-DINA from the data generated by G-DINA.

***gs* Level**	**Distribution**	**Sample size**	**EM_BN**	**GD_BN**	**IRP_BN**	**IRP_EM_BN**	**IRP_GD_BN**	**G-DINA**
*g*=*s*=0.2	Uniform	1,000	0.741	0.740	0.816	0.902	0.908	0.906
		500	0.350	0.307	0.829	0.896	0.898	0.898
	Mvnorm	1,000	0.736	0.660	0.831	0.916	0.921	0.933
		500	0.358	0.421	0.831	0.910	0.916	0.924
Mixed *gs* (0.3, 0.2, 0.1)	Uniform	1,000	0.740	0.714	0.757	0.880	0.887	0.885
		500	0.412	0.275	0.763	0.862	0.872	0.871
	Mvnorm	1,000	0.738	0.431	0.782	0.898	0.916	0.922
		500	0.371	0.287	0.696	0.894	0.898	0.907

**Table 6:** The standard error of PCR by BNs and G-DINA from the data generated by G-DINA.

***gs* Level**	**Distribution**	**Sample size**	**IRP_EM_BN**	**IRP_GD_BN**	**G-DINA**
*s*=*g*=0.2	Uniform	1,000	0.025	0.025	0.019
		500	0.032	0.024	0.030
	Mvnorm	1,000	0.014	0.027	0.019
		500	0.038	0.036	0.044
Mixed *gs* (0.3, 0.2, 0.1)	Uniform	1,000	0.027	0.021	0.024
		500	0.061	0.069	0.037
	Mvnorm	1,000	0.050	0.032	0.020
		500	0.030	0.036	0.050

**Table 7:** The standard error of AACR by BNs and G-DINA from the data generated by G-DINA.

***gs* Level**	**Distribution**	**Sample size**	**IRP_EM_BN**	**IRP_GD_BN**	**G-DINA**
*s*=*g*=0.2	Uniform	1,000	0.006	0.007	0.005
		500	0.002	0.008	0.009
	Mvnorm	1,000	0.006	0.006	0.005
		500	0.011	0.009	0.011
Mixed *gs* (0.3, 0.2, 0.1)	Uniform	1,000	0.008	0.007	0.007
		500	0.020	0.023	0.011
	Mvnorm	1,000	0.025	0.011	0.026
		500	0.008	0.060	0.067

The PCR and AACR by BN and G-DINA models from the data generated by G-DINA and the data generated by BN based on the G-DINA data and fraction data are shown in Table 8. And the standard errors of each PCR and AACR are given in parentheses. In the initial BN construction, the BN parameters were estimated by IRP–EM method, then the BN models with determined parameters were used to simulate two types of datasets for analyzing. It can be expected that the results of the simulated datasets analyzed by the IRP–EM method to be higher than that analyzed by the IRP–GD method. Thus, these data were not analyzed by IRP–EM method to avoid overrating the IRP–EM method. The BN–IRP–GD method and the G-DINA model were used to process these data. In the dataset generated by BN based on the G-DINA data, the PCR and AACR of BN-IRP-GD method was lower than the G-DINA model, and this result was similar to the dataset directly generated by G-DINA as shown in Table 6. These two datasets were essentially conforming to the ideal G-DINA model assumption. However, when empirical data is used to estimate the BN parameters and generate data based on this BN model, the simulated data pattern might violate the G-DINA assumption or at least does not conform to the assumption of the G-DINA model as ideally as the G-DINA generated data. The BN-IRP-GD method provides higher classification rate than the G-DINA model.

**Table 8:** The PCR and AACR by BNs and G-DINA from the data generated by G-DINA and the data generated by BNs based on the G-DINA data and Fraction data.

**DATA/model**	**PCR**		**AACR**	
	**GDINA**	**IRP_GD_BN**	**GDINA**	**IRP_GD_BN**
G-DINA-gen	0.701 (0.020)	0.658 (0.029)	0.931 (0.005)	0.919 (0.007)
BN-gen-based on-GDINA data	0.643 (0.015)	0.618 (0.008)	0.910 (0.046)	0.901 (0.008)
BN-gen-based on-Fraction data	0.502 (0.082)	0.670 (0.004)	0.850 (0.033)	0.908 (0.002)

## Real Data Example

**Figure 3:**
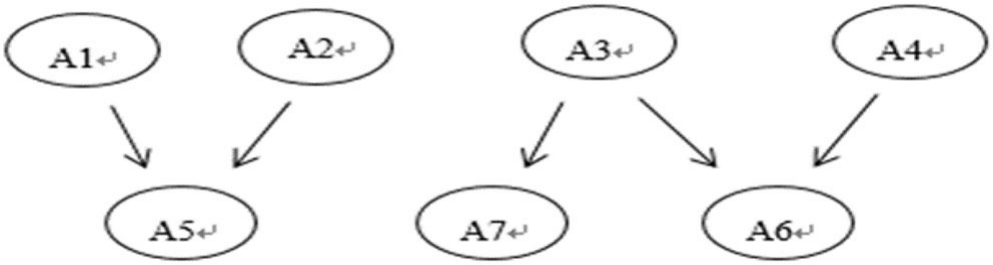
The attribute hierarchy relationship of the buoyancy.

To demonstrate the real-world applicability of the BN method, we used the dataset on the buoyancy concept developed by Gao et al. (2020) for cognitive diagnosis. These data have seven attributes, namely, (A1) know that the buoyancy direction is vertically upward, (A2) identify not only the gravity but also the buoyancy exerted on an object that is afloat, suspended, or immersed in liquid, (A3) know that the density is an object property, whose value is the mass divided by the volume, but still invariant to mass or volume changes, (A4) understand the meaning of a displaced liquid volume, (A5) calculate the buoyancy magnitude by analyzing the forces on objects, (A6) understand Archimedes' Principle, and (A7) decide whether objects will float or sink by comparing the object and liquid densities. These seven attributes have the hierarchy relationship as displayed in Figure 3. The Q-matrix with 14 items is shown in Table 9. A total of 1,089 eighth-grade students were chosen as subjects from 10 schools located in five east-coast cities in China. After excluding the subjects with blank test answers, 1,036 subjects remained. Two physics experts were invited to label the 50 randomly selected students on their mastery of the above attributes.

**Table 9:** The Q-matrix for buoyancy concept learning.

**Item/attribute**	**A1**	**A2**	**A3**	**A4**	**A5**	**A6**	**A7**
1	1	0	0	0	0	0	0
2	0	1	0	0	0	0	0
3	0	0	1	0	0	0	0
4	0	0	0	1	0	0	0
5	0	0	1	0	0	0	1
6	0	0	1	0	0	0	1
7	1	1	0	0	1	0	0
8	1	1	0	0	1	0	0
9	0	0	1	1	0	0	0
10	0	0	1	1	0	1	0
11	1	1	1	0	1	0	1
12	0	0	1	1	0	1	1
13	1	1	1	1	0	1	0
14	1	1	1	1	1	1	1

According to the attribute hierarchy relationships and the Q-matrix, we constructed a BN structure and trained the BN based on the IRP data first, and continued to estimate the BN parameters by GD method by regarding the previously estimated parameters as informative priors. The attribute patterns of the students could be predicted by BN based on the item response data. These data were also analyzed by the G-DINA model. From the previously selected 50 subjects, the estimated attribute patterns by the BN and G-DINA methods were compared with the labeling results obtained by the experts, and the PCR and AACR were calculated. Table 10 showed the agreement percentage of the BN and G-DINA analysis with the experts' labeling attribute patterns in the randomly selected 50 samples. We can see that the BN–IRP–GD method can achieve promising classification performance, and even higher than the G-DINA model in PCR. The agreement percentage between the BN and experts' labeling in each attribute was also a little higher than that observed between the G-DINA and experts' labeling. These results demonstrated the validity and feasibility of the BN–IRP–GD method.

**Table 10:** The agreement of BN or G-DINA analysis with the experts' labeling attribute patterns in randomly selected 50 samples.

	**A1**	**A2**	**A3**	**A4**	**A5**	**A6**	**A7**	**AACR**	**PCR**
G-DINA	0.96	0.96	0.92	0.94	0.88	0.76	0.88	0.90	0.50
BN	0.98	0.98	0.96	0.94	0.86	0.82	0.96	0.93	0.62

## Discussion

In this research, we conducted a simulation study comparing the parameter estimating algorithms in BN, including the EM method, the GD learning method, the IRP training method, and the combinations of IRP and EM (IRP–EM) or GD (IRP–GD) methods. The classification rates are considered as the performance indicators of the algorithms and the performance of G-DINA was adopted as a criterion to evaluate the performance of the improved parameter estimating method in BN. Real data analysis is followed to demonstrate the validity of the proposed method. The results show that the classification performances of the BN–IRP–EM and BN–IRP–GD methods are promising and even higher than the G-DINA model in certain conditions. The classification rates of EM and GD method are very low due to the reason that monotonic constraints may not be upheld. Moreover, both the EM and the GD methods are sensitive to the initial condition and liable to be trapped in a local optimality. We introduce the IRP training method to estimate the parameters of BN first, then continue to update the posterior values of the parameters by taking the previously estimated parameters as informative priors. The initial parameters estimated by IRP training are better starting values for EM and GD algorithms and can also solve the local optimality problems. The IRP data are constructed theoretically in every CDA and reflect a fair amount of test information. However, the IRP training method has no empirical information and the IRP–EM and IRP–GD methods can also solve these limitations and improve the classification rate of the IRP training method if be provided enough empirical samples.

This study demonstrated that, in the data generated by CDMs condition, the analysis by G-DINA was a little higher than the BN analysis based on the IRP–EM or IRP–GD method, which was as expected because the pattern of the data conformed to the assumption of G-DINA. However, in the data generated by BN based on the empirical data condition, the BN analysis based on the IRP-GD method outperformed the G-DINA analysis. But we cannot conclude that the BN methods are superior than the G-DINA model, just as we cannot claim that the G-DINA model are better than the BN methods in the first condition of the G-DINA simulated data. These two simulation conditions jointly demonstrated that the BN and G-DINA models each has their own advantages in the simulated data. From the above two conditions, we can see that the BN-data-generating method is more flexible. Different from the traditional CDMs, there are no explicit assumptions in BN. The parameters of BN reflect the patterns of the generated data and the parameters are determined by the data used to estimate the BN model. When the G-DINA data were used to estimate the parameters of the BN model, the generated data based on this BN model are conforming to the G-DINA assumption. Similarly, when the empirical data were used to estimate the parameters of the BN model, the generated data based on this BN model reflect the pattern of the empirical data, and these data do not necessarily satisfy the G-DINA assumption.

In the empirical study, the consistency of classification between BN and experts' judgment are higher than that between G-DINA and experts' judgment. Although both the average attribute classification rate and the pattern classification rate in BN are higher than the G-DINA model in this case, we are not intended to advocate that the BN model is more beneficial than the G-DINA model. These results are merely implying that the effectiveness of improved parameter-estimating method in BN is validated. In fact, the G-DINA model is a powerful framework that covers a wide variety of psychometric models, and through the comparison with the G-DINA, it demonstrates that if there are appropriate parameter-estimating methods, BN is also another great modeling framework that is compatible with many different types of data patterns in cognitive diagnostic assessment.

Shu et al. (2013) proposed to combine the NN trained by IRP with the traditional CDM to improve the performance of the traditional CDM. In their study, the IRP information cannot directly be used in traditional CDM, and the NN cannot incorporate the empirical information of samples. However, in BN, the parameter estimation can be accumulated according to the Bayesian theorem. First, the IRP and the corresponding attribute profile data are used to train the BN as a supervised learning method. Then the parameters obtained by the IRP training are regarded as the prior of the next stage of parameter estimation. When using EM or GD to continue the estimation, only the practical item responses are available, and the values of attributes are unknown and can be regarded as latent variable, thus this estimation stage is an unsupervised learning method. And the final parameters are the posterior probability according to the Bayesian theorem. An independent and complete model can accommodate the functions of both the NN and the traditional CDM (Shu et al., 2013). In other words, the supervised learning and unsupervised learning can be realized in the same model. From this perspective, the proposed method is a new progress.

BNs have been used extensively in the artificial intelligence community as student models for intelligent tutoring systems. The review article by Culbertson (2016) outlined many existing research studies on BN in educational assessment and it pointed that “BNs readily lend themselves to modular model building in which fragments of BN may be developed separately and combined freely based on a wide variety of types of relationships.” This aspect of a BN renders it a great potential for constructing the intelligent diagnostic assessment system to realize personalized learning. And this goal of realizing personalized learning is shared by CDA. But the BN can accommodate more node variables (e.g., Levy and Mislevy, 2004; Shute et al., 2008), which is more attractive to the educational assessment practice. In fact, in CDA, the MCMC method was used extensively for estimating the parameters of BNs (Wu, 2013; Almond et al., 2015; Levy and Mislevy, 2017). However, the MCMC method has some limitations. The first concern is the computational complexity and the efficiency, especially in cognitive diagnosis that needs immediate feedback of diagnostic information for instructions and learnings. Also, this method depends on the starting values to obtain stable and reliable estimates. The improved parameter-estimating method proposed in this article provides another approach to realize CDA based on BNs. And this method can be embedded into the diagnostic assessment system to achieve immediate feedback for personalized instruction. From the practical view, the optimization of the EM or GD method can provide more efficient computation compared to the MCMC method and can promote the CDA to be used in classroom assessment in which the instant feedback is needed. Moreover, due to the feature of modular model building and freely combining, BN can accommodate more attributes than traditional CDMs, which is needed for a practical diagnostic assessment system.

Naturally, we are aware of certain limitations in this study. The evaluation indicator of the BNs is relatively single and only the classification rate is adopted as the criteria of the BN's performance in this article, similar to what Sinharay (2006) described, “Model checking for BNs is not straightforward. For even moderately large number of items, the possible number of response patterns is huge, and the standard χ^2^ test of goodness of fit does not apply directly.” Sinharay (2006) applied the posterior predictive model checking (PPMC, Rubin, 1984) method to assess several aspects of fit of BNs applied in educational assessment. The PPMC method is a popular Bayesian model checking tool and combines well with the MCMC algorithms (e.g., Gelman et al., 2003). From the model comparison perspective, the most popular model fit indices are the AIC (Akaike, 1973) and BIC (Schwarz, 1978) criteria, which require knowing the number of free parameters to be estimated in the model. It is not so straightforward in complex BN model to count the number of parameters. Spiegelhalter et al. (2002) introduce a measure called DIC, which includes a Bayesian notion of dimensionality. But the DIC measure is also based on the MCMC algorithms. Thus, developing the new model fit indices of BN based on the IRP–EM or IRP–GD parameter-estimating method is a desired investigation area for future studies.

Additionally, Shu et al. (2013) combined the NN analysis based on the IRP data with the MCMC method to estimate the DINA model parameters in a small sample size. In traditional CDMs, the item parameters can be estimated to reflect the test quality. However, BNs have no straightforward item parameters to evaluate test quality. In future studies, we plan to apply the sensitivity analysis of BNs to determine the key attributes that influence each item response performance and the key items that determine each attribute diagnosis. Sensitivity analysis refers to an uncertain analysis technique to quantify the contribution of an observation node towards the uncertainty of the node of interest in BN. The sensitivity analysis results might provide insights into the knowledge structure and cognitive process of the students and assist in instruction design and personalized learning.

## Data Availability Statement

The raw data supporting the conclusions of this article will be made available by the authors, without undue reservation.

## Ethics Statement

The studies involving human participants were reviewed and approved by Academic Ethics Committee in Shenyang Normal University. Written informed consent to participate in this study was provided by the participants legal guardian/next of kin.

## Author Contributions

LW has made substantial contributions to propose the new algorithm, developed the R code, and wrote the first draft of the manuscript. TX and LW designed the research. YL collected the empirical data. YL and TX contributed to the final manuscript revision. All authors contributed to the article and approved the submitted version.

## Conflict of Interest

The authors declare that the research was conducted in the absence of any commercial or financial relationships that could be construed as a potential conflict of interest.
